# Applied global health diplomacy: profile of health diplomats accredited to the UNITED STATES and foreign governments

**DOI:** 10.1186/s12992-017-0316-7

**Published:** 2018-01-11

**Authors:** Matthew D. Brown, Julie N. Bergmann, Thomas E. Novotny, Tim K. Mackey

**Affiliations:** 1 National Institutes of Health, National Cancer Institute, Center for Global Health, Bethesda, MD USA; 2 Office of the Assistant Secretary for Health, U.S. Department of Health and Human Services, Washington, D.C., USA; 3 Department of Medicine, Division of Global Public Health, University of California, San Diego School of Medicine, San Diego, CA USA; 4 Global Health Policy Institute, San Diego, CA USA; 5 San Diego State University, Graduate School of Public Health, San Diego, CA USA; 6 Department of Family Medicine and Public Health, University of California, San Diego School of Medicine, San Diego, CA USA; 7 Department of Anesthesiology, University of California, San Diego School of Medicine, San Diego, CA USA

**Keywords:** Global health diplomacy, Health diplomacy, Foreign affairs, International relations, Health Attachés

## Abstract

**Background:**

Global health diplomacy (GHD) is a burgeoning field bridging the priorities of global health and foreign affairs. Given the increasing need to mobilize disparate global health stakeholders coupled with the need to design complex public health partnerships to tackle issues of international concern, effective and timely cooperation among state actors is critical. Health Attachés represent this coordination focal point and are key diplomatic professionals at the forefront of GHD. Despite their unique mandate, little is published about this profession and the perspectives of those who work in the field.

**Methods:**

Through purposive sampling, we performed in-depth qualitative interviews with seven Health Attachés: three foreign Health Attachés accredited to the United States and four U.S. Health Attachés accredited to foreign governments. Our interviews explored four key topics: the role and mission of Health Attachés, skills needed to perform GHD, examples of successes and challenges in accomplishing their respective missions, and suggestions for the future development of the diplomatic profession.

**Results:**

We identified several lessons to apply to the growing field of GHD. First, GHD actors need to receive appropriate training to successfully negotiate the intersection of global health and foreign affairs. Participants suggested several areas of training that would benefit GHD actors: diplomacy and negotiation, applied science, and cross-cultural competency. Second, participants articulated the need for a career path for GHD practitioners, increased opportunities for on-the-job training and mentored experiences, and GHD competencies with defined levels of mastery that can be used in occupational evaluation and career development.

**Conclusions:**

Our findings indicate that skills in diplomacy and negotiation, applied science, and cross cultural competency are essential for the statecraft of Health Attachés. Additionally, establishing a clear career pathway for Health Attachés is critical for future maturation of the profession and for fostering effective global health action that aligns public health and foreign diplomacy outcomes. Achieving these goals would ensure that this special cadre of diplomats could effectively practice GHD and would also better position Health Attachés to take the lead in advancing shared global health goals among nation states in a new era of twenty-first century diplomacy.

**Electronic supplementary material:**

The online version of this article (10.1186/s12992-017-0316-7) contains supplementary material, which is available to authorized users.

## Background

According to the World Health Organization, global health diplomacy (GHD) is an emerging field that bridges the disciplines of public health, international affairs, management, law, and economics, with a focus on negotiations that impact the global policy environment for health [1]. Global health, introduced by Koplan as an evolution of the term ‘international health’, refers to the multidisciplinary practice of tackling diseases of public health concern by partnering with nations around principles of health equity, rather than reinforcing national borders in protective isolation [2]. Achieving strategic national objectives through persuasion and attraction is a critical component in the practice of diplomacy, statecraft, and foreign affairs [3]. This is also reflected in the growing importance of health security, with today’s world more interconnected and mobile than ever, diseases of global significance cannot be tackled by countries acting in isolation [4].

The concept of nations joining together in the diplomatic fora to tackle public health problems is a core principle of GHD [2]. In this context, GHD is defined as having dual goals of improving health while strengthening relations among nations [5]. Today, GHD is viewed as a necessary tool in the practice of modern or “smart” diplomacy, expanding traditional spheres of economic, political, and military diplomacy [6]. The global response to the Ebola outbreak in 2014 is an acute illustration of the need for timely and effective practice of GHD [7]. Illustrating this need, among a list of missteps highlighted by several panels and committees set up to review the performance of the WHO during the outbreak, was a five-month delay in the declaration of a Public Health Emergency of an International Concern (PHEIC) and lack of coordination among WHO members states regarding travel bans, in clear violation of the International Health Regulations (IHR), both of which hampered response efforts by the global community and reflect the importance of effective diplomacy in emergency health situations [8, 9].

It should be noted that beyond the WHO definition, there are multiple forms of GHD described in the literature which have helped develop this nascent field [1, 10]. Katz et al. (2011), and Brown et al. (2014) described three levels of GHD, each with respective actors, tools, roles, and levels of accreditation: *core, multi-stakeholder, and informal* [1, 10]. *Core* GHD actors are officially accredited Health Attachés and diplomats, charged with representing and linking public health institutions in one government to public health institutions in another government, and are the primary subject of this study [1, 10]. Due to the formalized processes of credentialing Health Attachés, involving obtaining the agreement between two state Foreign Affairs Ministries, *core* GHD practitioners are the smallest number of GHD practitioners and employ tools primarily focused on state-level action. *Multi-stakeholder* GHD actors include government employees and multilateral representatives, who have more varied levels of credentialing, and thus represent a larger population of practitioners, and employ tools focused on multi-stakeholder action. Lastly, *informal* GHD, which includes host country officials, non-governmental organizations, private enterprises, universities, and the general public, have the fewest required credentials and subsequently represent the largest number of practitioners and most diverse set of associated tools [1].

GHD at each level of practice is equally important and necessary for effective global health action. Each distinct group cultivates respective best practices, definitions, tools, and has distinct comparative advantages that can be leveraged to influence diplomatic outcomes. Our study focuses on describing the perspectives of *core GHD* actors, primarily the cadre of Health Attachés, both because of their official credentialing status between state actors, and unique experiences serving as representatives of governmental public health institutions. Health Attachés represent core GHD practitioners that collect, analyze, and act on information concerning health in a foreign country or countries and cultivate relationships establishing critical links between public health and foreign affairs stakeholders and institutions [11]. To be effective, Health Attachés must represent the views of their governments and forge partnerships with other governments, multilateral institutions, private sector companies, non-governmental organizations, academia, and the public.

Despite their role as a focal point in GHD, Health Attachés have not been adequately described in the literature, and no profile has ever been published capturing their perspectives on the practice of GHD. Additionally, little is known about core GHD as a form of tradecraft, or the process of career preparation involved in becoming a Health Attaché. In fact, since there is no formalized training or preparation to become a Health Attaché, and many come with varied levels of foreign affairs knowledge or experience, traditional diplomats may not readily consider their critical roles within the Embassy or Mission. In response to this knowledge gap, we set out to understand the roles and responsibilities of currently-accredited Health Attachés, as well as describe perceived challenges they face in the field of GHD. Study findings can help develop more targeted training programs to better prepare diplomats, Health Attachés, or any similar professional charged with the practice of GHD, as they respond to public health emergencies and ultimately improve the ability of nations to find and cultivate common ground when tackling global health challenges.

## Methods

### Participant selection

This qualitative study utilized seven key informants’ interviews among foreign Health Attachés accredited to the United States, and U.S. Health Attachés accredited to foreign governments. The selection criteria and recruitment of Health Attachés began in 2013, with all interviews completed between 2013 and 2014, and was limited to U.S. government (USG) Health Attachés and foreign Health Attachés accredited to the U.S. Government. As stated in our study definition, Health Attachés are highly specialized core GHD practitioners representing the interests of state level actors and are few in number, and thus our inclusion criteria limited the number of potential study participants to currently practicing core GHD practitioners [12]. During the period of data collection, there were only 13 individuals eligible for inclusion in the study population. While not all agreed to participate, we were able to evaluate non-participants’ stated reasons for declining to participate. This study was approved by the Institutional Review Board at the University of California, San Diego and San Diego State University (Protocol #1538089).

#### Foreign Health Attachés

Our inclusion criteria for foreign diplomats included individuals who were officially accredited to the U.S. Government residing in the Washington, D.C. area, and who had the word “health” in their official diplomatic title as it appeared on the Diplomatic List, an inclusion criteria similar to what has been used in prior studies [1, 13]. Accredited foreign diplomats are diplomats whose credentials from their respective embassy or government have been accepted by the U.S. Department of State (DOS). This accreditation process, stipulated in the Vienna Convention on Diplomatic Relations (VCDR), allows diplomats to represent the views of their respective government to another government, as well as to the greater diplomatic community [14].

As stipulated in the VCDR, all accredited foreign diplomats are listed on a “Diplomatic List,” published regularly by the DOS, Office of the Chief of Protocol, on their publicly available institutional web page [15]. This list includes the individual’s name and address, diplomatic rank and formal title, which gives a brief indication of the diplomat’s area of specialization and function in the Mission [16]. The Mission refers to the government staff and constituent agencies that reside within the diplomatic buildings, residences, and compounds that make up the respective embassy community. We used the DOS Diplomatic List to perform content analysis of the characteristics of foreign Health Attachés and for recruitment of qualitative thematic analysis through interviews. The Diplomatic List published in Spring 2011 and Winter 2012 showed that only seven of the more than 177 countries have an accredited diplomat to the U.S. with the word “health” in their job title (Table 1) [16].

**Table 1:** Diplomats in Washington, D.C., accredited to the United States with the word ‘Health’ in their diplomatic title

	Country Embassy/Delegation	Diplomatic Title
1.	Canada	Counselor for Health
2.	Denmark	Health and Training Attaché
3.	European Union	Minister-Counselor for Food Safety
4.	France	Health and Consumer Affairs
5.	Kuwait	Health Attache
6.	Saudi Arabia	Health Attaché
7.	South Africa	Counselor for Health

#### U.S. Health Attachés

Eligible individuals included only U.S. diplomats assigned as Health Attachés abroad (at the time of data collection, only five countries had U.S. Health Attachés assigned: China, India, South Africa, Switzerland [Geneva, assigned to the U.S. Mission to the United Nations], and Brazil) [1, 12]. The U.S. Department of Health and Human Services (HHS), Office of Global Affairs (OGA) is the U.S. governmental unit responsible for assigning Health Attachés to U.S. Embassies abroad. U.S. Health Attachés have an interagency appointment process that engages the most impacted HHS agencies in the interview and selection process of the U.S. Health Attaché to be potentially selected and deployed to the country of assignment. This interagency appointment process allows the Health Attaché to represent all HHS agencies and the Secretary in an international context, enables them to report on health issues in the foreign country or region of assignment, and helps them to link public health agencies and other stakeholders between countries or regions of assignment. U.S. Health Attachés also provide scientific guidance to U.S. Ambassadors and other members of the U.S. Mission on areas of public health practice. They maintain key health-related relationships in the foreign country, and help support U.S. government responses to public health issues and challenges [12]. We contacted all five accredited U.S. Government Health Attachés for participation in our qualitative interviews for thematic analysis and all agreed to participate. Their countries or regions of assignment, diplomatic titles, and respective public health counterparts were reviewed for content analysis and are listed in Table 2.

**Table 2:** U.S. Health Attachés assigned abroad by the United States Department of Health and Human Services

	Country	Diplomatic Tile/Primary Counterpart
1.	Brazil	Health Attaché/Ministry of Health
2.	China	Health Attaché/Ministry of Health and Family Planning
3.	India	Health Attaché/Ministry of Health and Family Welfare
4.	South Africa	Health Attaché/Department of Health
5.	Geneva (in Switzerland)^a^	Health Attaché^a^/World Health Organization

^a^Health Attaché’s is assigned to the U.S. Mission to the United Nations, and counterpart is the World Health Organization, a multilateral organization, adopted from Paper presented at 141st APHA Annual Meeting (November2–November 6, 2013), http://apha.confex.com/apha/141am/webprogramadapt/Paper278367.html

### Study procedures

Given the small population eligible for this study, we contacted all eligible professionals who met our inclusion criteria. We conducted semi-structured interviews with all those who agreed to participate, recording and analyzing reasons for non-participation. Interviews were conducted face-to-face if possible, or administered over the phone when foreign travel was not practical. For participants located in the Washington D.C. area, interviews were arranged at a time and location convenient for the participant. For participants not located in the Washington, D.C. area, interviews were conducted over the phone at a time that was convenient for the research participant.

Informed consent was obtained from all participants and included permission to record and transcribe the interview. Interview notes were taken and incorporated into the narrative transcript. All participants were permitted an opportunity to review and clarify any part of the interview text before finalizing the transcript. Participant incentives were not offered in this study. Since all subjects’ names, titles, addresses, and respective country mission or embassy were publicly available, no promise of anonymity was possible, and while no names were reported in the analysis or reporting of results, no degree of anonymity was possible. Specifically, we identify all quotations and references to participants’ responses by only the embassy or country and the identity of the individual respondent is not included in the analysis, or the reporting of any results. These issues were covered in a consent form which was administered and consent obtained prior to starting the interview process.

A priori themes were drawn from the literature and used to develop the interview guide [1, 17, 18]. We focused on six domains related to the practice of GHD [19]. These domains represent gaps in the GHD literature and have not been previously described by practicing Health Attachés [20–22]. These domains included:

1. Health Attaché office and organizational structure, including their purpose, scope, and definition of GHD;
2. Activities and goals of their office;
3. Diplomatic challenges undertaken in achieving GHD-related goals;
4. Health-related activities that required diplomatic negotiations;
5. Specific or general training that is helpful in serving as a Health Attaché;
6. Suggestions to help improve the field and practice of GHD.

The complete interview guide is attached in Additional file 1.

### Data analysis

All audio-recordings were transcribed verbatim. Transcripts were uploaded into MAXQDA for analysis. The first author (MB) and a second author (JB) reviewed all notes and transcripts and participated in the coding process. Transcripts were first read and reviewed to develop an understanding of the content and to identify emergent topics/themes [23]. Open codes were created from these *emergent* themes [23, 24]. These themes were combined with our a priori codes, as derived from our review of the literature, and compiled to create a codebook. The codebook included a description of each code’s content, inclusion/exclusion criteria, and a text example. Transcripts were then coded separately by the first author and second author. After coding was completed, transcripts were reviewed for discrepancies, resolutions discussed, and the final codes were applied to the transcript of the interviews.

## Results

Of the thirteen Health Attachés eligible to participate in the study, both foreign and U.S. Government, seven agreed to be interviewed (Table 3). These included three foreign Health Attachés accredited to the United States and four U.S. Health Attachés serving at U.S. embassies abroad. The foreign Health Attachés were serving at the Canadian Embassy, the European Union (E.U.) Diplomatic Mission, and the Italian Embassy in the Washington, D.C. area. The U.S. Health Attachés were assigned to U.S. Embassies in South Africa, China, India, and Geneva.

**Table 3:** Eight Health Attachés accredited to the United States and five Health Attachés accredited to foreign governments, who participated in the global health diplomacy study, 2012–2014

	Country	Y/N	Interview Month/Year, or reason for refusal
Diplomats in Washington, D.C., accredited to the United States, with “Health” in Diplomatic Title			
1.	Canada	Yes	February 2012
2.	Denmark	No	Position vacant February 2012; referred to E.U. Health Attaché
3.	European Union	Yes	February 2012; also encouraged we interview Italy Science Attaché
4.	Italy	Yes	August 2014 (referred by the E.U. Health Attaché)
5.	France	No	Position did not address public health issues, referred to E.U. Health Attaché
6.	South Africa	No	Clinical provider of health services, does not address public health issues
7.	Saudi Arabia	No	Clinical provider of health services, does not address public health issues
8.	Kuwait	No	Clinical provider of health services, does not address public health issues
U.S. Health Attachés Assigned Abroad by the United States Department of Health and Human Services			
1.	Brazil	No	Unable to schedule and position became vacant during data collection
2.	China	Yes	March 2014
3.	India	Yes	February 2014
4.	South Africa	Yes	August 2014
5.	Geneva	Yes	October 2014

Several reasons were cited for lack of participation among the foreign diplomats. The Health Attachés in the South Africa, Saudi Arabia, Kuwait, Denmark, and France Embassies felt unqualified to participate in a study on GHD. South Africa, Saudi Arabia, and Kuwait each responded that they were not public health professionals, and their main roles were as medical practitioners who provided primary health care to diplomatic personnel stationed at the Embassy and to the families who reside in the Washington, D.C. area. In the case of Saudi Arabia and Kuwait, they also assisted expatriate citizens in navigating both health care and insurance access in the United States.

In the case of Denmark and France, these diplomats explained over the course of several emails and phone conversations that they felt unqualified to participate in an interview about GHD. They both explained that while their profiles included health, they primarily focused on management and reporting of economic and political affairs, rather than health affairs. Both independently suggested interviewing the Health Attaché for the E.U., who was already included as a study participant. Both diplomats independently asserted that the E.U. Health Attaché would be the most appropriate person to represent their countries as the E.U. Health Attaché supported the 28 respective E.U. Missions in the Washington, D.C. area regarding public health matters, and additionally was very active as a full-time Health Attaché within the diplomatic community.

During the interview with the E.U. Health Attaché, she suggested the inclusion of the Science Attaché for the Italian Embassy and the Canadian Health Attaché for study participation, as they were the most active among the foreign diplomatic community on public health issues in the Washington, D.C. area. The U.S. Health Attaché assigned to Brazil was unable to be interviewed due to staff turn-over and a resulting vacancy in the post during the study’s data collection period.

### The role and position of Health Attachés

*“I am the ambassador’s primary advisor on health issues and coordinating representational or policy related issues in the health sector.”* ***-- U.S. Health Attaché India***

All participants stated that their primary role was to act as the main advisor to the ambassador and the overall Mission on health matters in addition to managing health related activities. Most of the Health Attachés were solely part of a bilateral mission, meaning the purpose of the embassy is primarily to maintain a formal relationship, codified in agreements, between a sending and receiving government. However, in two cases, E.U. and Geneva, the Health Attaché was part of a multilateral delegation. As such, they represented their respective sending government to a multilateral institution. The U.S. Health Attaché in Geneva is part of the U.S. Mission to the United Nations, with a focus on the World Health Organization (WHO). The E.U. Health Attaché represents the European Union, and supports and interacts with the 28 E.U. country Missions’ resident in the United States, as well as represents the E.U. to the U.S. Government and the diplomatic community in the Washington, D.C. area. Both multilateral relationships are codified in formal agreements within their constituent institutions.

Interestingly, no Health Attaché was part of a dedicated health section in any Mission (Table 4). Instead they were embedded in other embassy sections, such as the Commercial (E.U), Economic (Canada), Science and Technology Section (Italy), Environment, Science, Technology and Health (ESTH) sections, or Political sections (U.S). The Health Attachés in the U.S. Embassies in China, India and South Africa, and the Canadian Health Attaché were the only participants who had their own employees and a unit dedicated solely to public health, rather than acting as a solo professional. Health Attachés without a dedicated health section had a direct reporting pathway to either the Ambassador, or the Deputy Chief of Mission. In the case of Canada, Italy, and the E.U., the Health Attaché reported through another section head of the Embassy. In Italy’s case, the Attaché reported to a Science and Technology Section head, in Canada’s case to the Economic Section Head, and the E.U. case to the Commercial Section head. However, all also maintained a reporting line to the Ambassador. It is interesting to note that as reported by the Geneva U.S. Health Attaché, in the U.S. DOS organizational structure of a Mission, if no ESTH section or officer is present, public health matters normally fall to the Economic Section, despite the Geneva Health Attaché sitting historically in the Political Section. However, in all cases, there was also a direct reporting relationship to their national public health authority in their home countries. This represents a double reporting line to Mission leadership (a primary reporting line) and a domestic public health agency/ministry (a secondary reporting line), reflecting the dual role in organizational structure and reporting responsibilities of Health Attachés.

**Table 4:** Foreign and U.S. Health Attachés reporting relationships and Section Size

	Country	Reporting Ambassador/Deputy Chief of Mission	Section	Direct Reporting to Public Health Agency	Number of Staff
Foreign Health Attachés in Washington, D.C., accredited to the United States					
1.	Canada	Yes	Econ	Yes	4
2.	E.U	No	Econ	Yes	4
3.	Italy	No	Science	Yes	2
U.S. Health Attachés assigned abroad by the United States Department of Health and Human Services					
4.	China	Yes	ESTH*	Yes	2
5.	India	Yes	ESTH*	Yes	2
6.	South Africa	Yes	ESTH*	Yes	3
7.	Geneva	Yes	Political	Yes	1

^*^Environment, Science, Technology and Health section

### What makes a Health Attaché?

As demonstrated in our literature review, GHD does not have standardized competencies as a field or required training for practitioners, and as such, no Health Attaché reported being trained to be a Health Attaché. As stated by the Canadian Health Attaché, “One of the deficiencies in global health diplomacy is not having a common language.” The Canadian Health Attaché lamented that the profession has to create its own best practices and common terminology while also identifying needed skills and gaps in training for successful careers. Participants voiced that there were four main skills needed for successful GHD practice: diplomatic and negotiation skills, public health and scientific knowledge, an understanding of their Mission’s overall priorities, and cross-cultural competency.

Health Attachés cited numerous specific skills developed during their respective careers that contributed to various professional successes while serving in a diplomatic Mission. The Italian Health Attaché highlighted the need for technical skills in the field of health.

*“[You need a] strong technical background. Sort of a professional diversification…because the real challenge is to understand what science means, to be able to read and understand…areas of interest by different scientists…”* ***—Italy Health Attaché***

The Health Attaché from the E.U. referred to the importance of negotiations skills when working on the Trans Pacific Partnership (TPP), a trade agreement among 12 Pacific Rim countries, which has undergone years of negotiations and is currently in a state of flux. She cited simulation games used to develop her negotiation skills as part of her career development.

*“Sometimes you have bad arguments, or you are sent into a mine field which you can't defend… we make a difference in our work with simulation games about negotiation so you [can test various] different outcomes.”* ***-- E.U. Health Attaché***

The U.S. Health Attaché to China highlighted the subtle professional skills and cultural understandings needed in diplomatic negotiations.

*“In international negotiations, it's all about postures. In addition to body language, listening, it's also cultural sensitivity and, of course, nobody can learn everything about every culture but instead of trying to put forward your position too quickly... even playing poker might help too.”* ***– U.S. Health Attaché China***

### Health diplomacy in action

To be effective in their roles, Health Attachés reported that they engage in daily activities of tracking existing and negotiating new agreements, organizing visits and attending meetings, drafting briefing documents, and meeting counterparts, collaborators, and other actors vested in public health issues in the country or region of assignment. As stated by one U.S. Attaché, all activities while serving as a Health Attaché require maintaining and building relationships, the bread and butter of diplomacy.

*“Relationship building is a key component of my responsibilities being the sole HHS representative in this country and this part of the world. The relationships are critical to my effective work and that’s a very important part of my job*.” ***– U.S. Health Attaché South Africa***

Health Attachés reported that they must be able to create and draw upon relationships within their host country to advance the priorities of their Mission while searching for an intersection of mutual interests, for both the sending and host governments. Part of building and/or maintaining these relationships comes from chairing and attending committees, working groups, or ad-hoc coordination efforts on health issues and collaborations in the country or region of assignment. Relationships built from these events are then utilized in meetings that manage or maintain requirements related to existing agreements, renew health agreements, and establish new partnerships, accords, or agreements codifying mutual requirements and benefits among parties.

Moreover, each Health Attaché reported employing knowledge gained from these relationships and priorities when contributing to internal planning documents with the Mission, or crafting briefing documents, memos, and talking points or speeches for Mission and governmental leadership. As the U.S. Health Attaché in Geneva mentioned, sometimes “we have a need for key elements to be respected, like human rights, inclusion of sexual and reproductive health services … provided to women and a range of things [that need to be included].” Since Health Attachés have established relationships with other country representatives, they reported being effective in identifying and exploiting areas of agreement and implementing mutually beneficial health initiatives, such as an innovation initiative supported by the Canadian Embassy in the below example:

*“That was a good example of global health diplomacy in that we’re providing seed money to these entrepreneurs, medical entrepreneurs in their own countries that would then spin off to a [business to] benefit the country.”* ***-- Canada Health Attaché***

Health priorities are also part of health portfolios with established goals and objectives of the Mission in the health sector, and are typically part of a larger Embassy strategic planning process, with several foreign policy goals. While most Mission plans are not available for public review or comment and remain unpublished, they are sometimes shared with government counterparts, and are often important tools to advocate for allocating resources, such as the U.S. Government’s President’s Emergency Plan for AIDS Relief (PEPFAR), which provides five-year strategic plans that in turn guide development of annual Country Operational Plans in collaboration with the host country [25, 26]. Another example is the European Union’s “Third Health Programme (2014–2020)” which involves all countries in the Union, and has objectives that impact global health. This plan, developed from a protracted and deliberate consensus-building process, has a health component to engage E.U. member states, universities, industry partners, and the general public, and is published online every six years [27].

Another example of a multi-year consensus process involving Member States engaged in GHD is the preparation process for resolutions for the World Health Assembly, the convening body of the WHO [28]. In describing one negotiation, the U.S. Health Attaché remarked:

*“This one ended up being about two years of negotiations; from 2010 to 2012 … we built a consensus around a public health approach to dealing with substandard and counterfeit medicines. We created a new member state mechanism which is effectively a subsidiary body of the World Health Assembly to kind of manage and oversee WHO engagement on counterfeits.”* ***-- U.S. Health Attaché to WHO Geneva***

One Mission priority, cited by all seven Health Attachés, was global health security. As the Italian Health Attaché commented:

*“We also tend to react on the spot by different interests depending on different priorities; Ebola these days [and] global health security is taking priority.”* ***-- Italy Health Attaché***

The participants stated that health security, found in most global health initiatives, requires continuous discussions with diplomats and other government officials as well as many representatives within the private sector. The Global Health Security Agenda (GHSA), is a multi-state collaborative that was initially launched by the United States in partnership with WHO and is now governed by a multi-country steering group that includes several international organizations as advisers. It also includes the participation of foundations, non-governmental organizations, and civil society, all with the common goal of accelerating implementation of the IHR [29]. Reflecting the importance of health security in the health diplomacy portfolio, Health Attachés from each of the seven countries were all active participants in multiple GHSA meetings and activities.

During the study data collection period, four GHSA events were held. These were located in Washington, D.C. in February 2014, Helsinki in May 2014, Jakarta in August 2014, and the White House in Washington, D.C. in September 2014 [30]. All seven participants attended one or more of these meetings and were involved in inviting and preparing attendees on health-related security issues. In the case of China, India, and South Africa, the diplomatic exchange also involved a *demarche* concerning attendance at a forthcoming GHSA meeting [31]. A *demarche* is a formal communication from one government, to another government, usually hosted through respective foreign ministries, and often involves a face-to-face meeting to ensure the context of the communication is clearly communicated.

### The future of global health diplomacy

All seven participants strongly believed that GHD as a field and practice would continue to expand and grow in importance in the future, and consequently must be supported and resourced accordingly. A challenge cited by India, South Africa, China, Geneva, Italy, the E.U. and Canada, was a lack of resources to maintain the requirements of their job, while at the same time managing increasing demand for their services and an increasing workload. The U.S. Health Attaché in South Africa succinctly summarized this point:

*“The amount of international health engagement continues to grow, whether that’s bilateral relations or mutual recognition of interests in Health Security, or just plain globalization [that] makes the world smaller, we are going to continue to do more things together…the amount of health diplomacy engagement required to manage and facilitate is a challenge.”* ***-- U.S. Health Attaché South Africa***

As asserted by all the participants, GHD is critical to ensuring the success of multi-state engagement on health issues.

All Health Attachés cited that as the practice and use of diplomatic activities involving health continue to grow, workloads likewise increase, but did not anticipate that resources would follow suit. Specifically, participants identified a lack of funding to hire additional staff, support travel, or host meetings or workshops. As stated, only India, China, South Africa, and Geneva had more than one employee in addition to the Health Attaché. Given this, they predicted that meeting the increasing demands of diplomatic activity in health will be a critical challenge to overcome in the future.

Finally, in addition to resource challenges, Health Attachés in India, South Africa, and China all cited that there is a perceived fundamental tension between the goals of public health, which requires tackling public health threats by partnering with nations around principles of health equity, involving shared responsibility and collective action, and those of foreign policy, which emphasizes national interests and often actions of protective isolation.

*“…they have very different priorities than we do or they have very different objectives than we do…that there is anybody at the table who has very different objectives than we do… I think that’s a problem. Certainly something that needs to have some sort of reconciliation…”* ***– U.S. Health Attaché India***

All participants cited that despite this tension, health continues to be present in nearly all foreign policy goals, be them commercial, trade, or security, and this will mean a greater need for negotiations by diplomats with competencies in health engagement within a foreign affairs environment.

## Discussion

As outlined in this study, Health Attachés are key practitioners and are poised to leave a significant impact on the current and future trajectory of GHD. Importantly, our findings can contribute to pedagogy, additional research, and refinement of training processes in the field of GHD. We collected information on best practices and identified challenges in GHD as experienced by *core* GHD practitioners. We found that Health Attachés are in a unique position to report on how diplomacy can be applied in the field of global health and foreign affairs as they act as the main advisers to diplomatic personnel regarding public health matters in both their sending and host countries. Essentially, they act as the link between governments on health issues, which requires them to be successful in building and maintaining relationships at all levels of GHD.

To successfully create and cultivate these relationships, Attachés need a unique set of cross cultural, multidisciplinary, and diplomatic skills and experiences that permit them to successfully identify and exploit areas of mutual agreement and resolve or minimize areas of disagreement among multiple stakeholders. They must be knowledgeable and effectively navigate various health and foreign policy issues (from intellectual property rights, international development aid flows, health security, health and human rights, social determinants of health, to subject specific issues such as the health risks of falsified or substandard medication and acute health emergencies such as Ebola or Zika) and also need to be nimble and quickly learn about emerging health and foreign policy challenges. They must also be able to carefully and attentively listen to concerns and converse with multiple actors on many subjects, while concomitantly understanding the cultural context of health issues in the country where they work. Finally, they must have some training and competencies in foreign affairs to understand how the various health topics fit within their Mission’s overall foreign policy goals and objectives [32–34].

Participants also discussed specific areas in which the field could be improved. For example, to facilitate communication among key actors, one suggested creating a key contact list of health professionals and their areas of specialization in a country or a region. Given concerns about limited resources for GHD, technology adoption (such as utilizing existing platforms of LinkedIn, WeChat, WhatsApp, etc.) may have the potential to facilitate greater communication among the public health and diplomatic community if properly resourced by an appropriate focal point in the host nation.

This technology adoption could also be integrated into formal credentialing process with the sending and receiving foreign ministries, effectively modernizing the current static Diplomatic List hosted by a county’s foreign ministry. Such a platform could assist GHD actors in knowing who to contact on specific health subjects and could serve as a dissemination platform of knowledge management for GHD practitioners. Respondents also suggested that, similar to GHSA and PEPFAR, sharing of planning documents (e.g. Mission public health planning or global health Mission strategic plan documents such as the E.U. strategic plan for health engagement) would be beneficial to the field of GHD by increasing transparency, accountability, and eliminate redundancy concerning health activities and priorities of partner governments. Similar models for this practice were described in PEPFAR’s 5-Year Strategic Country Plans, and annual Country Operational Plans, as well as the GHSA External Evaluations.

Additionally, Health Attachés also help write and shape Mission priorities. Both the line of communication and the direct involvement in shaping health priorities are critical points for leverage and integration of health goals in traditional areas of diplomacy. In this sense, Health Attachés can serve as important advocates for health initiatives if they fit appropriately with the Mission’s overall objectives. This takes on additional significance given that all participants reported that health issues are present in nearly all foreign affairs goals and objectives and that these needs are projected to grow over time. However, for GHD to continue growing in the future, GHD practitioners must be able to balance public health and foreign affairs goals and objectives and find strategic areas of overlap and convergence. Bridging global health and foreign affairs goals is a specialized practice and perspective, which Health Attachés are perfectly positioned to carry out on the front lines of GHD [1].

Participants also described a perceived tension between public health and foreign affairs goals. While not all Health Attachés agreed about whether public health goals should be used to advance foreign policy objectives, or if foreign policy objectives should be used to advance public health goals, participants did believe that a shared perspective is needed [35]. As the U.S. Health Attaché to India stated, neither field’s goals supersede the other; GHD practitioners should be working toward the same ends as a collective Mission. In part, given the lack of training and preparation needed to become a Health Attaché which is in stark contrast to foreign affairs practitioners, this tension may be attributed to the lack of standardization of defined practice competencies and differences in preparation between the fields of GHD and foreign affairs. GHD is unlike standard areas of traditional diplomatic practice, including political, economic, public affairs, and defense diplomacy, which all have well-established career paths, including practice competencies, defined levels of mastery, and extensive preparation needed to encumber a diplomatic posting at various levels. Standardizing the field and practice of GHD may help address and reconcile this tension and more effectively bring stakeholders in these oftentimes conflicting areas closer together. In alignment with this reported need for GHD professional skill building, our results also suggest that multidisciplinary global health training (where core competencies are currently being developed) could result in better-prepared Health Attachés who could more effectively conduct GHD [19, 36].

Finally, each participant stated that the best preparation and training for work in the Mission as a Health Attaché, is ‘on the job training’. Yet conversely, as stated, no Health Attaché was formally trained to be a Health Attaché. Increased investment in standardized pedagogy and more robust multidisciplinary training in combination with access to mentored experiences may better reinforce skill development in an applied context which could also contribute to a more well defined GHD career path. Resources to establish this type of training, including rigorous pedagogy, applied and measurable, need to be further developed. This step would help lead to a true development and expansion of a specialized cadre of core GHD practitioners, and ensure that Health Attachés have a defined career path and take a rightful place at the forefront of advocating for global health in twenty-first century diplomacy.

### Limitations

Our study’s main weakness was our limited sample of respondents and as such we did not explicitly separate results between USG and foreign Health Attachés. As reported, this study focused only on core GHD practitioners and this population of state actors is by definition very small in number. While we would have preferred to interview a greater number of foreign Health Attachés to better understand how their roles differ from those of U.S. Health Attachés, we only included diplomats who were responsible for health as indicated by their diplomatic title as listed on the Diplomatic List, which is the source of all core GHD practitioners.

Any study on core GHD practitioners should employ this sampling method as a baseline. Though future studies could build on this definition and expand the inclusion criteria (including potentially to multi-stakeholder and informal health diplomacy actors) and stratify results based on category/characteristics of Health Attaché, this was beyond the scope of the study objective given the primary focus to describe the experience of a core GHD practitioner. Our interviews were also completed in 2013–2014, limiting the generalizability and applicability of findings given subsequent changes in the political and global health policy environment that may influence health diplomacy and the practice and perceptions of Health Attachés.

Additionally, we were unable to travel for this study, and were unable to include health diplomats that may be posted in other countries (e.g., a Canadian Health Attaché posted in China). This limited our findings to Health Attachés with a United States nexus. Future studies could be designed to expand this sampling frame to other jurisdictions to better balance any potential location-based bias, and could also include case studies illustrating successes and challenges of Health Attachés in their respective careers to better illustrate thematic findings. Despite these limitations, the variety of participants each had a unique perspective, with many common points and shared experiences, that generalization to the larger population are appropriate, and provide a first in-depth qualitative analysis of *core* GHD actors, and as such is a critical contribution to helping mature and develop this expanding and critical field of practice.

## Conclusions

This is the first study to describe the practice of GHD as a tradecraft, from the perspectives of currently practicing Health Attachés, illustrating how these core health diplomats represent state actors and link public health and foreign affairs stakeholders towards more effective global health action. Our findings indicate that skills in diplomacy and negotiation, applied science, and cross-cultural competency are essential for the tradecraft of Health Attachés. Additionally, establishing a clear career pathway for Health Attachés is critical for future maturation of the profession and for fostering effective global health action that aligns public health and foreign policy outcomes. Achieving these goals would ensure that this special cadre of diplomats could more effectively practice GHD and would also better position Health Attachés to take the lead in advancing shared global health goals among nation states in a new era of twenty-first century diplomacy.

## Additional file

Additional file 1: Semi-Sutured Interview Guide for Profile of Health Diplomats Accredited to the United States and Foreign Governments. (DOCX 23 kb)

## Abbreviations

DOS: U.S. Department of State
EU: European Union
GHD: Global Health Diplomacy
GHSA: Global Health Security Agenda
HHS: U.S. Department of Health and Human Services
IHR: International Health Regulations
OGA: U.S. Department of Health and Human Services, Office of Global Affairs
PEPFAR: U.S. President’s Emergency Plan for AIDS Relief
VCDR: Vienna Convention on Diplomatic Relations
WHO: World Health Organization
