# Supplementary material for: Applied global health diplomacy: profile of health diplomats accredited to the UNITED STATES and foreign governments
Source: Global Health. 2018 Jan 11;14:2. doi: 10.1186/s12992-017-0316-7 (PMC5765610; doi:10.1186/s12992-017-0316-7)
Supplement: Additional file 1: — Semi-Sutured Interview Guide for Profile of Health Diplomats Accredited to the United States and Foreign Governments. (DOCX 23 kb) [file 12992_2017_316_MOESM1_ESM.docx]

**Appendix: Semi-Sutured Interview Guide for Profile of Health Diplomats Accredited to the United States and Foreign Governments**

**Project Title:**  Applied Health Diplomacy: Profile of Health Diplomats Accredited to the United States **Project Date:** 2013

**Method:** semi-structured, face-to-face interview

**Topic:** global health diplomacy

**Target Audience:** Health diplomats accredited to the U.S. Department of State

**Principal Investigator:**  Matthew Brown, [mbrown.cdc@gmail.com](mailto:mbrown.cdc@gmail.com)

### Instrument Title: Semi-Structured Interview Guide

*Below is a general guide that we will use to lead our individual interviews. In order to probe new or unanticipated issues, we may modify this guide as new information is learned from participants.*

*Before starting, conduct the informed consent process*

# I. Introduction

# Greet the participant and introduce yourself.

- Explain the general purpose of the interview discussion and why the participant was chosen.
- Obtain informed consent for participation in the research study and for recoding of the interview.
- Explain the presence and purpose of recording equipment.
- Address the issue of confidentiality – that country names will be collected, but individual names will be coded. Participant will get the opportunity to see the transcript of the interview and redact any portion of the text.
- Discuss interview can be paused if a break is needed.
- Invite the participant to introduce himself: name, title, government he represents and countries where s/he has served.
- Read the following study summary to the participant:

*This is a research study to determine how international diplomats accredited in the United States understand the concept of health diplomacy*

*Global Health Diplomacy has recently been embraced as an academic research topic and foreign- policy focus area. While there is much dialogue on Global Health Diplomacy, there remains a vague set of definitions and concepts that define this practice.*

*For the U.S. government, the Department of Health and Human Services has appointed an Assistant Secretary for Global Health, Dr. Nils Daulaire, and a new Office of Global Health Diplomacy in the DOS, led by Ambassador Eric Goosby.*

*However, the lack of a formal conceptual framework for the field of global health diplomacy and the undefined job description for a ‘health diplomat‘ calls for more information from those who practice diplomacy in this space. Professionals such as you and your colleagues can share experiences that will also inform future health diplomacy training efforts. .*

*This interview will ask about your conceptual understanding of global health diplomacy with regard to:*

1. *The purpose, scope, and definition of this practice*
2. *What activities are undertaken are within that rubric;*
3. *Diplomatic challenges faced in achieving GHD-related goals;*
4. *Health-related activities in which you were involved that required diplomatic negotiations;;*
5. *Specific or general training in GHD;*
6. *Suggestions to help improve the field and practice of GHD.*

# II. Interview

*A semi-structured interview will be conducted. Probe for least one specific example of each response.*

Please tell me about your:

1. Official duties and responsibilities
2. Portfolio of health-related activities or topics;
3. Any official organizational structure within your Embassy for managing health issues;
4. Mission and country specific health agenda and goals;
5. Other organizations and entities you collaborate with related to global health projects or negotiations;
6. Can you describe any relevant global health accomplishments or negotiations of which you have been part?
7. Have you had any specific training in global health diplomacy? Are there options for the training programs in which you have participated to include health diplomacy training?
8. What skills are important to have as a practicing health diplomacy – as a health attaché?
9. What is your definition of health diplomacy?
10. Do you think anything specific should be done to improve the practice of GHD?
11. Is there anything else you would like to add about GHD?
12. What is your own definition of GHD?

**End of Interview**

## **III. Closing**

*Closing remarks: That’s all the questions I have. Thank you for participating in this discussion. I will be back in touch within the next two weeks with a transcript of our interview, at which point you will be able to redact any portion of the text.*
